# Dual mediating effects of marital satisfaction and family decision-making power on the relationship between self-disclosure and depressive symptoms in IVF-ET/ICSI-ET patients: a cross-sectional study

**DOI:** 10.3389/fpsyt.2025.1538279

**Published:** 2025-04-24

**Authors:** Yuedi Jia, Jieyu Wang, Qianhua Xu, Guiying Luo, Ren Chen, Danni Wang

**Affiliations:** ^1^ Department of Health Promotion and Behavioral Sciences, School of Public Health, Anhui Medical University, Hefei, Anhui, China; ^2^ Reproductive Medicine Centre, The First Affiliated Hospital of Anhui Medical University, Hefei, Anhui, China; ^3^ School of Health Services Management, Anhui Medical University, Hefei, China

**Keywords:** assisted reproductive services, self-disclosure, marital satisfaction, family decision-making power, depression

## Abstract

**Objective:**

Infertility has become one of the major public health problems, and assisted reproductive technology is the main treatment. Depressive symptoms are one of the most common mental illnesses treated with this technology. The aim of this study was to investigate the effects of partner self-disclosure, marital satisfaction and family decision-making power on depressive symptoms in assisted reproductive therapy patients.

**Methods:**

A cross-sectional survey of self-disclosure, marital satisfaction, family decision-making power and depressive symptoms was performed in 1076 patients who underwent IVF/ICSI-ET treatment at the Reproductive Medicine Centre of the First Affiliated Hospital of Anhui Medical University.

**Results:**

(1) Age and power base were the influencing factors of depressive symptoms. (2) The results of mediation effect test showed that marital satisfaction had a significant suppressing effect on depressive symptoms in assisted reproductive therapy patients, accounting for 30.05% of the total effect; family decision-making power had a significant partial mediating effect on depressive symptoms in assisted reproductive therapy patients, accounting for 28.81% of the total effect.

**Conclusion:**

Marital satisfaction and family decision-making power play a partial mediating role between self-disclosure and depressive symptoms. The score of marital satisfaction helps to reduce depressive symptoms, but the increase of family decision-making power predicts the increase of depressive symptoms. In addition, the results highlight gender differences in marital satisfaction and family decision-making power and the complexity of family relationships.

## 1 Introduction

The World Health Organization (WHO) defines infertility as a disease of the reproductive system, characterized by the inability to conceive within a period of at least one year of unprotected sexual intercourse (1). Infertility has become a significant global public health concern, affecting an estimated 17.5% of the global adult population (2). In China, the prevalence of infertility among couples of childbearing age has reached 25%, representing a significant public health concern (3). Assisted reproductive technology (ART) represents one of the most efficacious methods of treating infertility (4). Nevertheless, while ART offers patients a glimmer of hope, it also presents a multitude of challenges. ART patients not only contend with the emotional distress and physical discomfort associated with infertility, but they also navigate the distressing experiences and financial burdens associated with ART treatments. *In Vitro* Fertilization and Embryo Transfer (IVF-ET) and Intracytoplasmic Sperm Injection-Embryo Transfer (ICSI-ET) are currently the most widely used ART modality in China, with a rate of 43.92% (5). This mainstream treatment modality includes invasive tests, surgical procedures, substantial medical costs (on average 30,000 CNY per cycle), and uncertainty surrounding the outcome of the treatment (6).

Depression represents one of the most prevalent psychological disorders among patients undergoing ART (7). Among ART patients, 16.5% of female and 5.8% of male partners report experiencing long-term depressive symptoms (8). In patients undergoing IVF-ET/ICSI-ET, the prevalence of depressive symptoms is 17.9% for females and 6.9% for male partners (9). This will have a direct impact on the individual health, marital status and family relations of the patients, and will result in serious negative consequences that are not conducive to the positive development of society (10–13). Therefore, it is essential to clarify the risk factors and related relationships of depression in IVF-ET/ICSI-ET patients, and timely intervention to control the prevalence of depression in this population and reduce the burden of disease in the future.

According to family systems theory, the family is an organized system in which individuals engage in dynamic interactions with one another (14). As the smallest unit of the family, the couple constitutes the only participants in the dynamic interactions within the household. Therefore, marital relationships directly impact family stability and resilience, which in turn affects individual mental health (15). Moreover, couples not only participate together in treatment but also face reproductive pressures and make decisions collectively, highlighting the importance of communication between partners. Currently, the majority of patients undergoing ART in China are young couples, with an average age of 31.0 ± 4.6 years (16). In this age group, the intimate relationships of couples are primarily influenced by modern romantic practices, emphasizing mutual disclosure in communication, where partners reveal their authentic inner feelings (17). Such disclosure enhances intimacy within the relationship and is a key factor for its success (18). Empirical studies have demonstrated that the frequency of self-disclosure is an effective predictor of relationship satisfaction and has a beneficial impact (19). A study on infertile patients showed that those with higher marital satisfaction had a lower incidence of depressive symptoms (11). In summary, effective family functioning and healthy marital relationships can withstand external pressures, thereby safeguarding the mental health of ART patients and reducing the risk of depression. In prior research (20), the roles of self-disclosure in romantic relationships regarding relationship uncertainty and satisfaction were examined. However, the exact mechanism between self-disclosure, relationship satisfaction, and depression in patients experiencing ART is unclear and relevant empirical studies are lacking, and further research is needed.

Factors influencing family stability encompass not only self-disclosure but also the internal power dynamics within the family. According to resource theory, individuals who possess the most resources to meet the needs and goals of family members hold greater power within the family (21). The core of resource theory lies in individuals’ preferences, interests, and goals. Marriage and family life offer opportunities to achieve specific objectives, and as long as both parties perceive the cost-benefit ratio as favorable, these relationships are likely to endure (22). Scanzoni categorizes power into three dimensions: power bases (the resources possessed by spouses), power processes (interactions within the family, such as self-disclosure), and power outcomes (the final decision-makers in family matters, referring to decision-making authority) (23). Family decision-making authority encompasses the rights of members to make decisions on key issues, including choices related to production methods, housing construction or purchase, and the acquisition of major tools or high-end goods. This authority reflects an individual’s level of power within the family and their ability to control family resources (24, 25).

Individuals experiencing infertility are unable to conceive in the typical manner, limiting their access to essential reproductive resources. The loss of fertility may undermine the fundamental functions of the family and negatively impact marital stability. As a result, couples may feel their efforts are futile, leading to the marginalization of the infertile partner within the family. These changes can trigger emotional upheaval. For those facing infertility, a lack of power and status often creates internal pressure, diminishing self-esteem and self-worth, and ultimately resulting in negative emotions (26, 27).

The distribution of power within families has been confirmed as a significant predictor of marital satisfaction. Research indicates that higher levels of self-disclosure, decision-making authority, and marital satisfaction are closely associated with positive relationship outcomes (21). Additionally, a study found that decision-making power within families negatively impacts the well-being of women with lower educational attainment and social status (28). These findings suggest a correlation between family rights, marital satisfaction, and depression. However, the relationship between decision-making authority in IVF-ET/ICSI-ET patients and marital satisfaction remains unverified, necessitating further investigation to clarify this relationship’s nature. Therefore, this study aims to explore the relationship between self-disclosure and depressive symptoms while analyzing the potential mediating roles of decision-making authority and marital satisfaction in this context.

## 2 Methods

### 2.1 Design and participants

This cross-sectional study was conducted at the Assisted Reproduction Centre of a tertiary care hospital in Hefei. Data were collected during the treatment assessment phase from 14 July 2021 to 3 October 2022 using a convenience sampling method. In order to be eligible for inclusion in the study, volunteers were required to meet the following criteria: (1) undergoing IVF/ICSI-ET treatment (including husbands who come to accompany their wives for treatment); (2) free of diseases that may affect sexual function; (3) free of psychiatric medications that interfere with sexual life; and (4) capable of reading, writing and understanding Chinese. Since the information survey was conducted in the treatment waiting hall, in order to protect the privacy of patients, this study used a paperless questionnaire with less information displayed at a single time on the mobile phone screen. Reduces the likelihood that patient information will be exposed to others. We will give each participant a free one-time body composition examination as compensation for their voluntary participation in this study. The Ethics Committee of Anhui Medical University agreed and approved to carry out this study with the ethics approval number 20200961 and informed consent of all patients. To ensure data reliability, we excluded any information that did not include age (n=10), couple information (n=266) and repeated questionnaires (n=46). A total of 1,076 patients agreed to participate and complete the questionnaire (response rate was 76.97%), of which 624 (57.99%) women and 452 (42.01%) male partners completed the questionnaire.

### 2.2 Measures

#### 2.2.1 Sociodemographic and clinical features

The self-reported questionnaire included information on socio-demographic factors and clinical characteristics. These included age, sex, annual income of the self and partner, education of the self and partner, cause and type of infertility, and duration of treatment. In this study, in order to examine the decision-making power of the family, the rights base of the couple’s rights (i.e., the difference in the couple’s annual income and the difference in the couple’s level of education) will be controlled in the regression equation. This study employs the absolute value of the grade difference in the couple’s annual income and the grade difference in the couple’s level of education, which is included as a control variable in the regression equation. See **Table 1**.

**Table 1:** Comparison of scores of various scales with different demographic characteristics.

Variables (coded)	Total	Depression		Statistic	*P*
		No (<15)	Yes (≥15)		
	(n = 1076)	(n = 752)	(n = 324)		
Age, Mean ± SD	30.96 ± 4.38	31.22 ± 4.52	30.35 ± 3.97	t=3.18	**0.002**
Infertility year (years), Mean ± SD	2.15 ± 2.30	2.09 ± 2.27	2.31 ± 2.36	t=-1.42	0.156
Infertility treatment time (mouths), Mean ± SD	26.77 ± 26.86	26.78 ± 27.45	26.75 ± 25.48	t=0.01	0.989
**Self-disclosure, Mean ± SD**	1.12 ± 0.61	1.10 ± 0.65	1.17 ± 0.52	t=-1.80	0.072
**Marital satisfaction, Mean ± SD**	1.62 ± 0.51	1.68 ± 0.45	1.49 ± 0.61	t=4.98	**<0.001**
**Family Decision Making Power, Mean ± SD**	0.00 ± 2.28	-0.38 ± 2.16	0.89 ± 2.31	t=-8.43	**<0.001**
**Depression symptoms, Mean ± SD**	11.46 ± 5.67	8.74 ± 4.13	17.78 ± 3.19	t=-38.91	**<0.001**
Gender, n (%)				χ²=0.86	0.353
Female (0)	624 (57.99)	443 (58.91)	181 (55.86)		
Male (1)	452 (42.01)	309 (41.09)	143 (44.14)		
Educational level, n (%)				χ²=36.46	**<0.001**
Primary and below (1)	141 (13.10)	75 (9.97)	66 (20.37)		
Junior high school (2)	230 (21.38)	167 (22.21)	63 (19.44)		
High school/technical secondary school (3)	222 (20.63)	142 (18.88)	80 (24.69)		
Junior college (4)	251 (23.33)	181 (24.07)	70 (21.60)		
Undergraduate course (5)	226 (21.00)	182 (24.20)	44 (13.58)		
Master degree or above (6)	6 (0.56)	5 (0.66)	1 (0.31)		
Spouse’s education level, n (%)				χ²=14.03	**0.015**
Primary and below (1)	32 (2.97)	26 (3.46)	6 (1.85)		
Junior high school (2)	271 (25.19)	185 (24.60)	86 (26.54)		
High school/technical secondary school (3)	172 (15.99)	125 (16.62)	47 (14.51)		
Junior college (4)	257 (23.88)	165 (21.94)	92 (28.40)		
Undergraduate course (5)	296 (27.51)	209 (27.79)	87 (26.85)		
Master degree or above (6)	48 (4.46)	42 (5.59)	6 (1.85)		
Educational class gap, n (%)				χ²=23.61	**<0.001**
0	378 (35.13)	293 (38.96)	85 (26.23)		
1	426 (39.59)	292 (38.83)	134 (41.36)		
2	182 (16.91)	108 (14.36)	74 (22.84)		
3	70 (6.51)	48 (6.38)	22 (6.79)		
4	19 (1.77)	10 (1.33)	9 (2.78)		
5	1 (0.09)	1 (0.13)	0 (0.00)		
Annual income (Yuan), n(%)				χ²=80.58	**<0.001**
< 10,000 (1)	186 (17.29)	165 (21.94)	21 (6.48)		
10,000−30,000 (2)	353 (32.81)	275 (36.57)	78 (24.07)		
30,000−60,000 (3)	102 (9.48)	52 (6.91)	50 (15.43)		
> 60,000 (4)	435 (40.43)	260 (34.57)	175 (54.01)		
Spouse’s annual income, n (%)				χ²=5.49	0.139
< 10,000 (1)	195 (18.12)	131 (17.42)	64 (19.75)		
10,000−30,000 (2)	203 (18.87)	154 (20.48)	49 (15.12)		
30,000−60,000 (3)	260 (24.16)	185 (24.60)	75 (23.15)		
> 60,000 (4)	418 (38.85)	282 (37.50)	136 (41.98)		
Income class gap, n (%)				χ²=15.92	**0.001**
0	414 (38.48)	273 (36.30)	141 (43.52)		
1	345 (32.06)	233 (30.98)	112 (34.57)		
2	253 (23.51)	202 (26.86)	51 (15.74)		
3	64 (5.95)	44 (5.85)	20 (6.17)		
Infertility reason, n (%)				χ²=10.98	**0.012**
Male factor (1)	186 (17.29)	125 (16.62)	61 (18.83)		
Female factor (2)	414 (38.48)	307 (40.82)	107 (33.02)		
Couple factor (3)	299 (27.79)	212 (28.19)	87 (26.85)		
Unexplained (4)	177 (16.45)	108 (14.36)	69 (21.30)		
Infertility diagnosis, n (%)				χ²=0.07	0.794
Primary infertility (1)	591 (54.93)	415 (55.19)	176 (54.32)		
Secondary infertility (2)	485 (45.07)	337 (44.81)	148 (45.68)		

t, t-test; χ², Chi-square test; SD, standard deviation;

The income class gap is the absolute value of the difference in income levels (coded) between spouses; the education class gap is the absolute value of the difference in education levels (coded) between spouses. The main study variables of this study are bolded.

#### 2.2.2 Depression

The World Health Organization defines depression as a common mental disorder characterized by a prolonged depressed mood or a loss of pleasure and interest in activities (29). Depressive symptoms were quantified using the Patient Health Questionnaire-9 (PHQ-9). The PHQ-9 was developed by Robert L. Spitzer at Columbia University in the 1990s to assess depressive symptoms over the past two weeks using diagnostic criteria from the Diagnostic and Statistical Manual of Mental Disorders (Fourth Edition) (DSM-IV). The PHQ-9 comprises nine items, each with four categorical options. The scoring principle is as follows: never = 0, a few days = 1, more than half the time = 2, almost every day = 3, from which the total score is calculated. The diagnostic scores of 5, 10, 15 and 20 represent the thresholds for mild, moderate, severe and very severe depressive symptoms, respectively (30). The scale has been employed by scholars across the globe, including those based in China, and has been demonstrated to be both reliable and valid. The Cronbach’s α coefficient for the PHQ-9 in this sample was 0.895.

#### 2.2.3 Family decision-making power

Family decision-making power is conceptualized based on the Major Family Affairs Decision Theory proposed by Tao Chunfang and Jiang Yongping (25). This theory views the authority to make significant family decisions as a symbol and true embodiment of familial power. Possessing this power signifies a position of authority within the family and control over family resources. This study utilizes data from the China Family Panel Studies (CFPS), a nationally representative large-scale survey, to assess family decision-making power through nine specific questions (31). The questions included the following: “You can decide on the daily expenses of the family”, “You can decide on the purchase of high-class goods/large agricultural machinery”, and so on. The respondents were also presented with the option of determining who would assist them with their children. Additionally, they were given the opportunity to choose whether to purchase and construct a residence, to invest or take out a loan, to purchase personal high-end goods, and to purchase a house. Furthermore, respondents were presented with the option of purchasing personal high-end goods, studying or working abroad, or supporting their parents. The value 0 represents a negative response, while the value 1 represents a positive response. The study then aggregates the scores of these nine variables to construct a composite score of the household’s decision-making power, which ranges from 0 to 9. A higher total score signifies an increased level of decision-making power within the household. The Cronbach’s alpha coefficient for the sample was 0.866. Furthermore, principal component analysis (PCA) was employed to extract the principal components from the aforementioned nine variables. The extracted indicator, designated as Decision, exhibited a correlation of up to 1.00 with the composite score of family decision-making power. Consequently, Decision was selected for the subsequent empirical analyses.

#### 2.2.4 Marital satisfaction

Marital satisfaction is defined as the degree to which an individual is satisfied with his or her marriage. In order to assess this, we employed a combination of two questions from the Family module of the China General Social Survey 2006 (CGSS2006). The CGSS represents the earliest national, comprehensive, and continuous academic survey project in China. The questionnaire items are highly representative of the population under study. The respondents were asked to indicate their level of satisfaction with their marital life following the receipt of treatment. The rating scale was as follows: dissatisfied = 0, more satisfied = 1, and very satisfied = 2. The question was as follows: “If you had the opportunity to choose your spouse again, would you choose the same one?” The scoring principle is as follows: a response of “wouldn’t” is assigned a value of 0, a response of “don’t know” is assigned a value of 1, and a response of “would” is assigned a value of 2. The scores for each item were summed and averaged to obtain a marriage satisfaction score, which ranges from 0 to 2, with higher scores indicating greater satisfaction. The sample’s Cronbach’s alpha coefficient was 0.702. Because the Kaiser-Meyer-Olkin (KMO) value of only two items must be 0.5, the factor loading coefficient is explained, and the factor loading coefficient was 0.878 (using Varimax).

#### 2.2.5 Self-disclosure

Self-disclosure refers to the act of individuals sharing their true inner feelings with their partners and is a fundamental aspect of intimate relationships (19). The China General Social Survey (CGSS) is the first large-scale, continuous social survey in China (32, 33). This study utilizes two questions from the CGSS 2006 questionnaire that reflect self-disclosure in the context of marriage to measure this phenomenon. The study examines self-disclosure among infertile couples after treatment, using the following two questions: “After treatment, my spouse listens to my concerns” and “After treatment, my spouse shares his/her concerns with me.” Responses were scored on a three-point scale, where 0 indicates “never,” 1 indicates “often,” and 2 indicates “occasionally.” Higher scores reflect greater levels of self-disclosure. The average of these two items was used for data analysis, resulting in a score range from 0 to 2. The Cronbach’s alpha for the sample was 0.744. The factor loading coefficient was 0.892 (using Varimax).

#### 2.2.6 Common method bias

In this study, the questionnaire completed by infertility patients was tested for common method bias using Harman’s one-way test. Five factors with eigenvalues greater than 1 were extracted from the results of the unrotated exploratory factor analysis. The first of these explained 27.977% of the total variance, which is less than 40%. Therefore, there is no evidence of common method bias in this study.

### 2.3 Data analysis strategy

Descriptive statistics and one-way analyses of the study data were completed using the Storm-based statistical platform (www.medsta.cn/software) and R version 4.3.0 (2023-04-21). The use of ChiPlot (https://www.chiplot.online/) enabled the performance of intra-group correlation analyses (the calculation of Pearson’s correlation coefficients between study variables) and the generation of heat maps. Potential mediation was analyzed with SPSSAU 24.0 (https://www.spssau.com), and mediation was tested by the bootstrap method (model IV). The level of significance was set at α = 0.05.

## 3 Results

### 3.1 Characteristics of participants

The effective sample size of this study was 1076 cases, of which 57.99% were female. The female participants were aged between 21 and 44 years, with a mean age of 30.96(S D=4.08) years. The male participants were aged between 22 and 53 years, with a mean age of 31.48(SD=4.73) years. According to the recommendation of the study (34), the PHQ-9 score greater than or equal to 15 was considered as depression, that is, severe and above. The total detection rate of significant depressive symptoms was 30.11%, 29.00% in women and 31.63% in men. The remaining baseline data are shown in **Table 1**.

Differences in scores between men and women for key variables are shown in **Table 2**. Comparing men and women, it was found that men had higher scores in marital satisfaction, family decision-making power, and depressive symptoms, while there was no difference in self-disclosure.

**Table 2:** Gender differences in the main variables.

Variables	Total (n = 1076)	Women (n = 624)	Men (n = 452)	Statistic	*P*
Self-disclosure, Mean ± SD	1.12 ± 0.61	1.12 ± 0.62	1.12 ± 0.59	t=0.05	0.964
Marital satisfaction, Mean ± SD	1.62 ± 0.51	1.54 ± 0.54	1.73 ± 0.44	t=-6.39	**<0.001**
Family Decision Making Power, Mean ± SD	0.00 ± 2.28	-0.41 ± 2.13	0.57 ± 2.36	t=-6.97	**<0.001**
Depression symptoms, Mean ± SD	11.46 ± 5.67	11.06 ± 5.87	12.02 ± 5.36	t=-2.79	**0.005**

t, t-test.

SD, standard deviation.

#### 3.1.1 Correlation analysis of couple’s rights-based, self-disclosure, marital satisfaction, family decision-making power and depression symptoms in infertility patients

**Figure 1:**
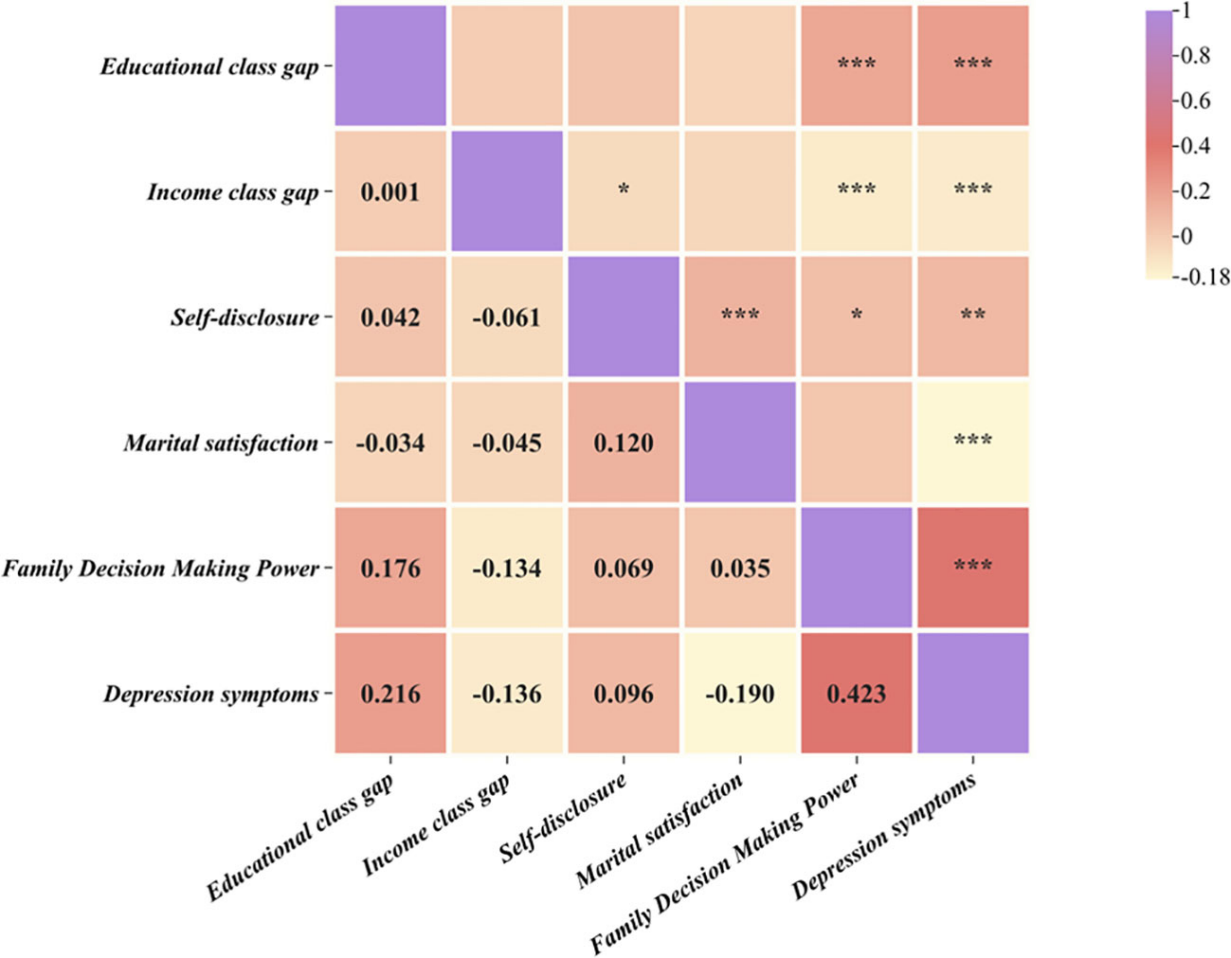
Correlation analysis of research variables (n=1076) ^*^ *P*< 0.05, ^**^ *P*< 0.01, ^***^ *P*< 0.001.

**Figure 1** presents the results of the correlation analysis between couple’s rights-based, self-disclosure, marital satisfaction, family decision-making power, and depressive symptoms in infertile patients.

The results demonstrated that educational class gap was positively correlated with family decision-making power, and depressive symptoms (*P*< 0.001), income class gap was negatively correlated with family decision-making power, and depressive symptoms (*P*< 0.001), and positively correlated with self-disclosure (*P*< 0.01), self-disclosure was positively correlated with marital satisfaction, family decision-making power, and depression (*P*< 0.001, *P*< 0.05, *P*< 0.01), marital satisfaction was negatively correlated with depressive symptoms (*P*< 0.001), and family decision-making power was negatively correlated with depressive symptoms (*P*< 0.001). The correlation between family decision-making power and marital satisfaction was not statistically significant.

### 3.2 Multivariate linear regression analyses predicting depression symptoms

As shown in **Table 3**, the results of multiple linear regression analysis are provided. Model 1 only included the general demographic and treatment variables, and model 2 included the study variables on the basis of model 1, and the *Adjust-R ^2^* is 0.256. VIF values are close to 1, and there is almost no multicollinearity, which can be used for regression analysis.

**Table 3:** Multivariate linear regression analyses predicting depression symptoms in the whole sample.

	Model1						Model2					
	*B*	S.E.	*p*	*β*	VIF	Tolerance	*B*	S.E.	*p*	*β*	VIF	Tolerance
Intercept	0.200	0.220	0.362	–			0.358	0.198	0.071	–		
Age	-0.025**	0.007	**<0.001**	-0.108	1.098	0.911	-0.021**	0.006	**0.001**	-0.092	1.104	0.906
Infertility year	0.013	0.015	0.391	0.029	1.357	0.737	-0.002	0.013	0.888	-0.004	1.378	0.725
Infertility during time	-0.002	0.001	0.213	-0.043	1.370	0.730	-0.001	0.001	0.363	-0.028	1.378	0.726
Gender	0.148*	0.060	0.014	0.073	1.023	0.977	0.078	0.056	0.164	0.039	1.109	0.902
Educational class gap	0.208**	0.030	**<0.001**	0.203	1.018	0.983	0.135**	0.028	**<0.001**	0.132	1.049	0.953
Income class gap	-0.035	0.030	0.248	-0.034	1.009	0.992	-0.033	0.027	0.224	-0.032	1.011	0.989
Infertility reason	0.092**	0.019	**<0.001**	0.145	1.047	0.955	0.056**	0.017	**0.001**	0.088	1.065	0.939
Infertility diagnosis	0.068	0.061	0.266	0.034	1.067	0.937	0.030	0.055	0.587	0.015	1.069	0.935
Self-disclosure							0.083**	0.027	0.002	0.083	1.028	0.973
Marital satisfaction							-0.217**	0.027	**<0.001**	-0.217	1.077	0.928
Family Decision Making Power							0.380**	0.028	**<0.001**	0.38	1.103	0.907
*R* ^2^	0.083						0.264					
*Adjust-R* ^2^	0.076						0.256					
*F*	*F* (8,1067) =12.057, *p<0.001*						*F* (11,1064) =34.705, *p<0.001*					
△*R* ^2^	0.083						0.181					
△*F*	*F* (8,1067) =12.057, *p<0.001*						*F* (3,1064) =87.298, *p<0.001*					

Dependent variable, Depression symptoms; The results of linear regression variable assignment have been presented in **Table 1**.

*p<0.05 **p<0.01.

### 3.3 Dual mediating roles of marital satisfaction and family decision-making power in self-disclosure and depressive symptoms

**Figure 2:**
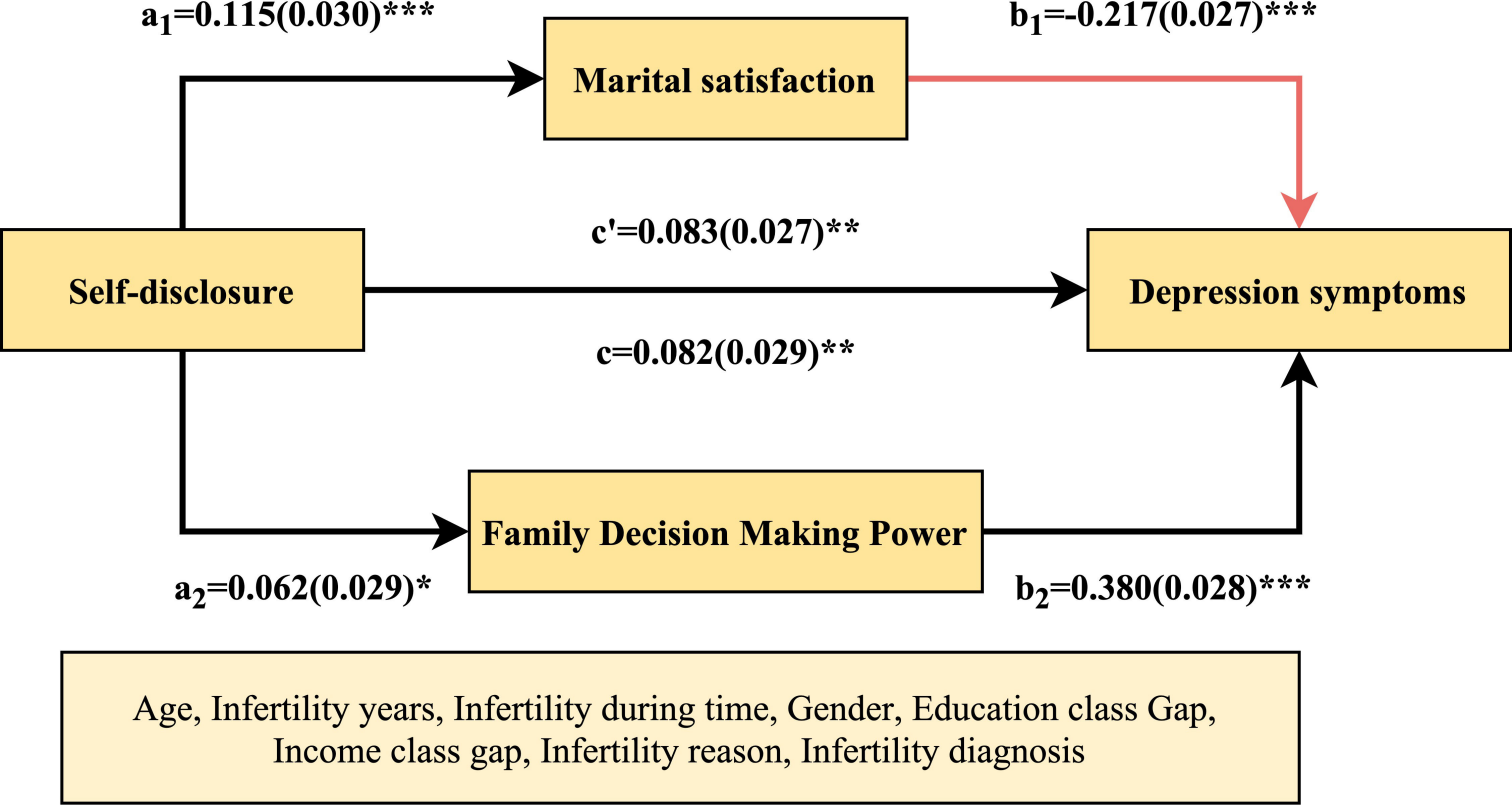
The mediating role of marital satisfaction and family decision-making power. In the study sample, the masking effect of marital satisfaction on self-disclosure and depressive symptoms (upper) and the mediating effect of family decision-making power on self-disclosure and depressive symptoms (lower), covariates (bottom) and standardized coefficients were reported, with standard errors in parentheses. ^*^ *P*< 0.05, ^**^ *P*< 0.01, ^***^ *P*< 0.001.

Three distinct regression models were constructed, each controlling for a set of confounders. These included age, gender, educational class gap, income class gap, infertility reason, infertility year, and infertility diagnosis. The first model was constructed using independent variables (self-disclosure) and dependent variables (depression symptoms). The second model was constructed using independent variables (self-disclosure) and mediating variables (marital satisfaction and family decision-making power) for regression model construction. The third model was constructed using an independent variable (self-disclosure), a mediator variable (marital satisfaction and family decision-making power), and a dependent variable (depression symptoms) for regression model construction. A repeated random sampling procedure was employed to draw 5,000 bootstrap samples (n=1076) from the original data set. The 95% confidence intervals for the mediating effect were estimated using the 2.5th and 97.5th percentiles. The mediating effect was deemed significant if the 95% confidence interval for the indirect effect did not include zero. The mediating effect was deemed significant if the 95% confidence interval for the indirect effect did not include zero. The results indicated that marital satisfaction was found to be a competitive mediation, while family decision-making power was found to be a complementary mediation, with effect sizes of 30.045% and 28.806% respectively, for the relationship between self-representation and depressive symptoms. In addition, comparing the M →Y path, and taking the absolute value of all the negative values in the confidence interval of the path coefficient, it is found that the confidence interval after the absolute value treatment has no overlapping part, and b_2_>b_1_, so family decision-making power has a greater impact on depressive symptoms than marital satisfaction. The mediation model diagram and path coefficients are shown in **Table 4** and **Figure 2**.

**Table 4:** Test of intermediary effect of couple relationship on family power and depressive symptoms.

Item	Symbol	Meaning	Effect	95% CI		*SE*	*z / t*	*P*	Conclusion	Effect Ratio
				Upper	Lower					
Self-disclosure →Marital satisfaction →Depression symptoms	a_1_*b_1_	Indirect effect	**-0.025**	-0.041	-0.011	0.008	-3.283	**< 0.001**	Suppressing effect	30.045%
Self-disclosure →Marital satisfaction	a_1_	X →M	0.115	0.057	0.173	0.030	3.879	**< 0.001**		
Marital satisfaction →Depression symptoms	b_1_	M →Y	-0.217	-0.271	-0.164	0.027	-7.956	**< 0.001**		
Self-disclosure →Depression symptoms	c'	Direct effect	**0.083**	0.031	0.135	0.027	3.118	**0.002**		
Self-disclosure →Depression symptoms	c	Total effect	**0.082**	0.024	0.139	0.029	2.782	**0.005**		
Self-disclosure →Family Decision Making Power →Depression symptoms	a_2_*b_2_	Indirect effect	**0.024**	0.000	0.046	0.012	2.012	**0.044**	Partial mediating effect	28.806%
Self-disclosure →Family Decision Making Power	a_2_	X →M	0.062	0.004	0.119	0.029	2.112	**0.035**		
Family Decision Making Power →Depression symptoms	b_2_	M →Y	0.380	0.326	0.434	0.028	13.764	**< 0.001**		
Self-disclosure →Depression symptoms	c'	Direct effect	**0.083**	0.031	0.135	0.027	3.118	**0.002**		
Self-disclosure →Depression symptoms	c	Total effect	**0.082**	0.024	0.139	0.029	2.782	**0.005**		

In the table, Boot standard errors, lower bound Boot CI, and upper bound Boot CI refer to the standard errors, lower and upper 95% confidence intervals of the indirect effects estimated using the bias-corrected percentile bootstrap method, respectively; all values are rounded to three decimal places. Standardized coefficients are reported. "→" represents the direction of the regression path, as shown in **Figure 2**.

## 4 Discussion

This study investigates the relationship between self-disclosure and depression in infertile couples, as well as the mediating roles of marital satisfaction and family decision-making power. Results indicate a significant association between self-disclosure and depression, with marital satisfaction and family decision-making power serving as partial mediators. However, no correlation was observed between family decision-making power and marital satisfaction.

The findings of this study indicate a correlation between age and power base and the occurrence of depressive symptoms. The proposition that patients of an advanced age are less prone to depressive disorders can be corroborated within the context of ecosystem theory. Ecosystem theory posits that the interactions between individuals and their environment are instrumental in shaping their developmental trajectory and adaptability (35). In this context, older individuals may encounter a greater number of environmental changes and challenges, which could potentially lead to the development of greater experience and resilience over time. The rights-based nature of couples’ rights is associated with depressive symptoms, which is consistent with the findings of previous research in this area (36). A comprehensive review of the literature reveals a clear correlation between the size of the education gap and the income gap and the prevalence of depressive symptoms. This phenomenon can be explained by the immersion cost effect, a concept derived from economic theory. China currently has a large population base and, in relative terms, higher education is more costly, including in terms of human, material and financial resources. A significant investment of resources does not necessarily yield the anticipated benefits, which may intensify the pressure on the individual (37). Given that couples are highly aware of each other, this imbalance is further increased by the comparison of the two partners, which in turn gives rise to and exacerbates depressive symptoms. It is also important to note that patients who are under pressure to have children and to undergo treatment may be compelled to disrupt their normal work patterns or even to cease employment in order to have children. This has the effect of increasing the disparity in the couple’s basic rights and reducing the total family income, thereby intensifying the pressure when treatment fails, which may in turn increase depressive symptoms (38, 39). This once again highlights the important role of the rights base in the emotions of assisted reproduction patients.

A comparison between males and females reveals that there are no significant differences in terms of depression (PHQ-9≥15) and self-disclosure. However, there are notable differences in depressive symptom scores, decision-making power within the family, and marital satisfaction. These differences may be related to gender behavioral disparities and traditional views. In traditional Chinese families, males are typically seen as the primary decision-makers, often regarded as the heads of the household. The inability to conceive not only challenges a man’s dignity but also contravenes traditional filial piety. Consequently, when faced with reproductive issues, men often avoid communication and may even conceal or distort facts to protect their dignity. Research indicates that the prolonged existence of such situations leads to a neglect of men’s psychological needs, trapping them in a state of learned helplessness (40). In contrast, women are more likely to seek out social resources, such as social support, to alleviate their emotional distress (41). However, studies have shown that support from husbands often does not satisfy wives, prompting women to seek support from other social networks, such as peer groups (42). The findings related to self-disclosure further confirm that the marital relationship, as the smallest unit of the family, involves interaction within the household. Nevertheless, couples undergoing IVF-ET/ICSI-ET experiences currently do not fulfill their genuine needs through their interactions. Furthermore, according to family resource theory, greater resources within a family correspond to greater power. In our study sample, the prevalence of infertility attributed to females is significant, which may result in women having fewer resources than men, consequently leading to lower family decision-making power.

The findings indicate a correlation between self-representation and depression, and suggest that marital satisfaction and family decision-making power may act as mediators between self-disclosure and depressive symptoms. Specifically, marital satisfaction and family decision-making power were found to act as both competitive and complementary partial mediation of self-disclosure and depressive symptoms. The conclusion of competitive and complementary partial mediation is based on statistical estimates rather than inherent psychological mechanisms. Nevertheless, no correlation was identified between marital satisfaction and family decision-making power.

Marital satisfaction serves as a competitive partial mediation in self-disclosure and depressive symptoms. One potential explanation is that self-disclosure as a mechanism through which couples engage in activities that foster marital bonding and facilitate stress release when confronted with significant challenges in their schedules or during the course of treatment (43). According to family systems theory and resource theory, couples in therapy tend to interact when faced with fertility - and life-related challenges. Such self-disclosure may serve to enhance intimacy, provide support, or facilitate catharsis and relief, thereby fostering a greater sense of intimacy and mutual trust between partners, which in turn contributes to greater satisfaction with the marital relationship, a finding that aligns with those of previous research (20). Concurrently, this enhancement in marital satisfaction serves to insulate patients from adverse influences pertaining to childbearing and general life circumstances, thereby attenuating the prevalence of depressive symptoms (44).

The role of family decision-making power as a complementary partial mediation between self-disclosure and depressive symptoms is a significant area of interest within the field of psychological research. One potential explanation is that the transition from arranged marriages to more autonomous unions in Chinese culture has led to a greater emphasis on communication in modern marriages. This has gradually encouraged couples to express themselves more freely within the relationship (45). Nevertheless, the degree of influence an individual exerts within the household is contingent upon their level of contribution. Typically, the greater the resources an individual possesses, the more they are able to contribute to the household (46–48). In terms of the resources available to couples, it can be observed that those who have greater access to resources tend to be more active in their marriages (21). It was therefore demonstrated that the frequency of self-representation was a predictor of the amount of decision-making power within the family unit. In accordance with the Iron Law of Responsibility proposed by K. Davis, the correlation between power and responsibility is direct. Consequently, the greater the authority to make decisions within a family unit, the greater the pressure on the individual. A high-pressure environment is thus more likely to result in depression.

The findings of this study suggest that self-disclosure has a direct and indirect influence on marital satisfaction and depressive symptoms. Based on the standardized coefficients, it can be concluded that while self-disclosure predicts marital satisfaction, family rights are a more significant predictor of depressive symptoms. Although previous studies have yielded mixed results regarding the role of family and marital relationships in the mental health of infertile patients, the majority of studies have indicated that these relationships are indeed significant (7, 15, 49–52). Nevertheless, there has been a paucity of cross-sectional studies examining couple communication and family rights, and the underlying mechanisms of their structure remain poorly understood, particularly in developing countries. The impact of self-expression on marital satisfaction and the reduction of depressive symptoms is less pronounced than that of family decision-making power on depression. Consequently, in clinical and intervention studies, it is imperative to consider not only the impact of the couple relationship on patients’ mental health but also the influence of the individual’s decision-making role within the family and their resource allocation strategies on mental health. Notably, this study did not find a relationship between marital satisfaction and family decision-making power, which is inconsistent with previous findings (21). One possible explanation is that when it comes to fertility issues, husband and wife act as one. At this time, couples increase intimacy through communication, and make decisions and solve problems in the communication process. At this time, family decision-making power does not directly affect marriage satisfaction, but adjusts marriage satisfaction and family decision-making power to affect emotions through self-disclosure. This suggests that the marriage pattern of ART couples is different from that of normal couples, and its scenario is more complex, which needs further study. Therefore, in future research and clinical practice, we should pay attention to the balance between husband and wife, help patients have a correct understanding of treatment and the relationship between husband and wife, and adjust their mentality. In particular, we should pay attention to the influence of cultural differences and consciousness cognition on patients’ psychology (28). Medical participants should pay attention to the communication between patients and their spouses, correctly guide self-disclosure between patients and their spouses, and create a healthy relationship between husband and wife, so that patients can get social support by revealing their difficulties in the family, have a better sense of security, and improve family resilience (15), so as to have a better relationship and mentality between husband and wife, so as to reduce patients’ depressive symptoms.

While our study enhances the current understanding of patients’ mental health by clarifying the relationships between various variables, it is essential to recognize that it is a cross-sectional study, which imposes limitations on causal inference. Therefore, longitudinal studies or experimental designs should be carried out to explore the causal relationship between the two items in the future. Secondly, the results of the data analyzed in this study demonstrated that although the masked effect of marital satisfaction accounted for a greater proportion of the effect, its effect size was still smaller than that of family rights. This suggests that there are additional influences or mediators between self-disclosure and depressive states, beyond the factors that were studied in this research. Meanwhile, the two variables used in this study, marital satisfaction and self-disclosure, were studied with only two items, and although the structural requirements were met, fewer items may not fully capture these structures. Future studies can use more accurate questionnaires to verify and explain the results. Despite our efforts to control for the basis of rights between couples, this was not a comprehensive approach. Future studies could be conducted in pairs or as couples to further explore the impact of different family rights models on the mental health of patient couples. Furthermore, the study population was from a single hospital in China, which imposes selection bias and limits extrapolation. Validation in different geographies and populations is recommended. This study’s short scale primarily focused on patients’ self-reported self-disclosure and marital satisfaction, overlooking other important communication behaviors and dimensions of marital quality. Future research needs to explore these aspects, such as facial expressions, body language, and personality compatibility between couples, especially in paired studies centered on couples. Such investigations will deepen our understanding of the connections between communication behaviors and evaluations of marital quality, ultimately promoting healthier relationships. Furthermore, it is essential to investigate additional influencing factors to enhance the model, ascertain the impact of various factors on mental health, elucidate the underlying causes of changes in mental health among infertile patients, and identify more practical and efficacious clinical interventions to assist patients in attaining physical and mental wellbeing.

Therefore, based on the above studies, this study suggests that there may be more complex family relationships in the infertile group that influence individual mental health status. It was also postulated that marital satisfaction and family decision-making power in infertile patients could act as mediators between self-disclosure and depression, influencing the direct effect of self-disclosure on both dimensions of depression. The potential mechanisms between self-disclosure and depression in patients undergoing assisted reproduction treatment provide new ideas for clinical research and intervention development.

## 5 Conclusion

Patients undergoing IVF-ET/ICSI-ET often experience depressive symptoms attributed to the challenges of infertility and the stresses associated with treatment. Our findings indicate that the prevalence of depressive symptoms among individuals undergoing IVF-ET/ICSI-ET is associated with both age and power base. We identified gender differences in marital satisfaction and family decision-making power, highlighting the complexity of family relationships. Additionally, patients’ self-disclosure has both direct and indirect effects on depressive symptoms through its impact on marital satisfaction and family decision-making power. This study enhances our understanding of the interplay between depressive symptoms and self-disclosure in populations receiving IVF-ET/ICSI-ET treatment. Therefore, healthcare professionals should consider not only the marital status of patients undergoing IVF-ET/ICSI-ET but also the dynamic changes in family power distribution to help alleviate depressive symptoms.

## Data Availability

The raw data supporting the conclusions of this article will be made available by the authors, without undue reservation.
